# The transcriptomic signature of responses to larval crowding in *Drosophila melanogaster*

**DOI:** 10.1111/1744-7917.13113

**Published:** 2022-10-17

**Authors:** Juliano Morimoto, Marius Wenzel, Davina Derous, Youn Henry, Herve Colinet

**Affiliations:** ^1^ School of Biological Sciences University of Aberdeen Aberdeen United Kingdom; ^2^ Programa de Pós‐graduação em Ecologia e Conservação Universidade Federal do Paraná Curitiba Brazil; ^3^ Institute of Mathematics King's College University of Aberdeen Aberdeen United Kingdom; ^4^ CNRS, ECOBIO (Ecosystèmes, biodiversité, évolution)—UMR 6553 University of Rennes Rennes France; ^5^ Department of Ecology and Evolution University of Lausanne Lausanne Switzerland

**Keywords:** intraspecific competition, larval crowding, life‐history, trade‐offs, transcriptomics

## Abstract

Intraspecific competition at the larval stage is an important ecological factor affecting life‐history, adaptation and evolutionary trajectory in holometabolous insects. However, the molecular pathways underpinning these ecological processes are poorly characterized. We reared *Drosophila melanogaster* at three egg densities (5, 60, and 300 eggs/mL) and sequenced the transcriptomes of pooled third‐instar larvae. We also examined emergence time, egg‐to‐adult viability, adult mass, and adult sex‐ratio at each density. Medium crowding had minor detrimental effects on adult phenotypes compared to low density and yielded 24 differentially expressed genes (DEGs), including several *chitinase* enzymes. In contrast, high crowding had substantial detrimental effects on adult phenotypes and yielded 2107 DEGs. Among these, upregulated gene sets were enriched in sugar, steroid and amino acid metabolism as well as DNA replication pathways, whereas downregulated gene sets were enriched in ABC transporters, taurine, Toll/Imd signaling, and P450 xenobiotics metabolism pathways. Overall, our findings show that larval crowding has a large consistent effect on several molecular pathways (i.e., core responses) with few pathways displaying density‐specific regulation (i.e., idiosyncratic responses). This provides important insights into how holometabolous insects respond to intraspecific competition during development.

## Graphical Abstract

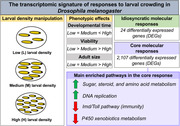

## Introduction

Intraspecific competition is an important evolutionary force leading to local adaptation, niche expansion and speciation through adaptive radiation (Grant, 1972; Werner & Sherry, 1987). Theoretical models have predicted—and empirical work has highlighted—that adaptation to novel resources is a key mechanism through which intraspecific competition affects niche expansion. For instance, theory predicts that high intraspecific competition in one niche or resource can lead to the invasion of underexploited niches by maladapted phenotypes (Wilson & Turelli, 1986) and that intraspecific competition modulates the degree of individual resource specialization within populations (Svanbäck & Bolnick, 2005). Supporting evidence for these models comes from work in *Drosophila melanogaster*, where populations experiencing high intraspecific competition levels adapt to cadmium‐containing diets (a toxic compound) more rapidly than populations experiencing low intraspecific competition levels (Bolnick, 2001). Similar patterns have been found in other species including tadpoles (Levis *et al.*, 2020), sticklebacks (Svanbäck & Bolnick, 2007), bees (Fontaine *et al.*, 2008) and beetles (Agashe & Bolnick, 2010), although in the latter species the pattern is less clear (Parent *et al.*, 2014).

In holometabolous insects, intraspecific competition is likely to be the strongest at the larval stage because of the underlying assumption that insect larvae are often much less mobile than adults (see e.g., Jones, 1977; Hansen *et al.*, 1984; Markow, 2015, but also Goddard *et al.*, 2020) and reliant primarily upon female oviposition site choices for feeding (Thompson, 1988; Larsson & Ekbom, 1995; Gripenberg *et al.*, 2010). In this case, intraspecific competition at the larval stage can trigger positive and negative responses that can carry‐over to adult stage and modulate fitness. On the one hand, high intraspecific competition at the larval stage beyond natural levels (“overcrowding”) increases stress‐tolerance through hormesis‐like responses (Henry *et al.*, 2018; Lushchak *et al.*, 2019), which may result in longer adult lifespan (Shenoi *et al.*, 2016). Moreover, experimental evolution studies in *D. melanogaster* have also found that populations adapt to higher intraspecific competition at the larval stage by increasing thermal tolerance, feeding rates and tolerance to toxic compounds (e.g., urea) (Bubli *et al.*, 1998; Borash *et al.*, 2000; Mueller *et al.*, 2005; Shakarad *et al.*, 2005), which can be interpreted as a plausible proximate explanation for the diet niche expansion in high intraspecific competition populations (Bolnick 2001). Similar effects have been described in other drosophilids (Nagarajan *et al.*, 2016). On the other hand, overcrowding constrains the availability of nutrients *per* *capita*, thereby constraining larval growth as well as adult morphology, behavior and fitness (Klepsatel *et al.*, 2018; Than *et al.*, 2020). In *D. melanogaster*, larvae developing in overcrowded conditions have unfavorable phenotypes that resemble those of individuals in protein‐deficient larval diets (Klepsatel *et al.*, 2018), suggesting that overcrowding strengthens the competition for nutrients and limits the availability of protein, which is essential for larval growth and adult reproduction. Moreover, overcrowding is also known to modulate the bacterial composition of the diet in which larvae are feeding which can affect pathogenicity of substrate, larval behavior, and life‐history traits (Wong *et al.*, 2017; Henry *et al.*, 2020). Similar effects were described in the tephritid fruit fly *B. tryoni* (Nguyen *et al.*, 2019) and other insects (reviewed by Than *et al.*, 2020). Although these examples highlight that physiological responses to crowding induce a range of phenotypic changes that can facilitate or hinder the survival and adaptation to local conditions, we know very little of the global gene expression patterns that underpin the responses to overcrowding. Previous target studies have examined molecular responses using only few candidate genes (Henry *et al.*, 2018; Lushchak *et al.*, 2019; Borash *et al.*, 2000), which precludes our understanding of broad gene expression profiles that shape the entire suite of phenotypic responses to overcrowding. For example, Henry *et al.* (2018) and Sørensen & Loeschcke (2001) found higher relative expression of *Hsp70* and/or *Hsp40*, which is a sign of oxidative stress, in overcrowded conditions. Oxidative stress modulates a range of physiological responses that influence life‐history traits, but we do not know the knock‐on effects of higher oxidative stress on broader physiological processes. As a result, we lack a proper understanding of pleiotropic constraints and physiological trade‐offs that can affect plastic and evolutionary responses to overcrowding and can in turn, modulate life‐histories, niche expansion and ecological specialization (Kawecki, 2008; Poisot *et al.*, 2011; Remold, 2012).

Here we address this knowledge gap by identifying for the first time the molecular basis of larval responses to overcrowding in a controlled experiment. We manipulated the intraspecific competition at the larval stage of *D. melanogaster* individuals using three larval density treatments (Henry *et al.*, 2018; Henry *et al.*, 2020) and investigated the global transcriptomic response to overcrowding. We used three larval crowding levels, one of which is within the natural range of larval crowding observed in nature and two crowding levels which are beyond the larval crowding found in nature, providing us with the opportunity to explore the molecular responses to extreme crowding (Morimoto & Pietras, 2020). *Drosophila melanogaster* is the ideal model system to investigate the molecular responses to overcrowding for several reasons. First, *Drosophila* larvae are thought to be relatively immobile and, therefore, strongly influenced by interspecific competition (Markow, 2015; Morimoto & Pietras, 2020). Second, larval density modulates a range of fitness‐related traits in both positive (e.g., hormesis‐like) and negative (e.g., lower mating success) manners (Borash *et al.*, 2000; Henry *et al.*, 2018; Lushchak *et al.*, 2019; Henry *et al.*, 2020). Third, responses to overcrowding modulate the strength of selection and can have intergenerational effects in *D. melanogaster* groups (Morimoto *et al.*, 2016; Morimoto *et al.*, 2017). Lastly, responses to crowding are involved in increased larval competitive abilities that can result in niche expansion in evolutionary experiments in this model (Bolnick, 2001; Shenoi *et al.*, 2016). Table 1 shows our predictions for the highest density treatment based on the literature.

**Table 1:** Predictions for the effects of larval crowding

Predictions	Literature
Downregulation of metabolic pathways linked to dietary protein (e.g., TOR) due to protein limitation caused by high larval density (Prediction 1)	Klepsatel *et al.* (2018)
Upregulation of immunity due to density‐dependent prophylaxis observed in other insects as well as upregulation of pathways related to survival (e.g., autophagy) if density‐dependent responses increase survival (Prediction 2)	Schmid‐Hempel (2003); Siva‐Jothy *et al.* (2005); Shenoi *et al.* (2016); Kapila *et al.* (2021)
Upregulation of stress‐related pathways (e.g., heat‐shock proteins) as an hormesis‐like response to overcrowding (Prediction 3)	Henry *et al.* (2018); Lushchak *et al.* (2019)
No change or downregulation of pathways involved in reproduction (e.g., spermatogenesis) although some insects respond to high larval density by increasing testis sizes relative to body mass	McGraw *et al.* (2007); Johnson *et al.* (2017); Than *et al.* (2020); Morimoto *et al.* (2021)

## Materials and methods

### Larval density manipulation and adult phenotyping

We used an outbred laboratory population of *D. melanogaster* established from wild individuals collected in September 2015 in Rennes, Brittany (France). Fly stocks were maintained, and all experiments conducted, at 25 °C and 70% relative humidity (12 h light : 12 h darkness) on standard food comprising inactive brewer's yeast (MP Bio 029 033 1205, MP Bio, 80 g/L), sucrose (50 g/L), agar (Sigma‐Aldrich A1296, 10 g/L) and supplemented with anti‐mold Nipagin (Sigma‐Aldrich H5501; 10% 8 mL/L). Adult flies from rearing stocks were allowed to lay eggs for less than 12 h on standard food supplemented with extra agar (15 g/L) and food dye. Eggs were collected with a paint brush, counted on moistened fabric and then transferred into new vials (50 mL; diameter = 23 mm) with 2.0 mL of standard food. We created three larval density treatments known to show contrasting phenotypes: L (low density; 5 eggs/mL), M (medium density; 60 eggs/mL), and H (high density; 300 eggs/mL) (see Henry *et al.*, 2018, 2020). Egg manipulation was performed identically in all treatments and we standardized time spent under the stereomicroscope to 15 min to eliminate handling effects on the experiment. Eggs were allowed to hatch and larvae were allowed to develop until the wandering stage (third‐instar larvae). At this precise point, we collected three replicates of 10 larvae per density treatment from independent vials (*N_larvae_ =* 90; *N_replicates_* = 9) into cryotubes, which were snap‐frozen in liquid nitrogen and stored at −80 °C. Due to the lack of sex‐specific phenotypic markers such as size and coloration (as seen in adults), larvae were selected without consideration of sex. We kept some vials unsampled (20, 2, and 2, respectively, for conditions L, M, and H) and allowed the flies to complete development until the adult stage in order to assess the viability, the development time, and the sex ratio. This was done to avoid any small sampling effects on the crowding of the developing larvae. Recently emerged adults (<24 h) were placed in single‐sex vials with fresh food for three days before freezing at −20 °C. Thirty males and 30 females per larval density treatment were then randomly collected, dried for one week at 60 °C and weighed in a microbalance (Mettler Toledo UMX2, Mettler Toledo, Greifensee, Switzerland; accurate to 1 μg). We examined effects of larval density on adult weight using generalized linear models (GLM) with Gaussian distribution followed by Student–Newman–Keuls post hoc tests using the *agricolae* package (De Mendiburu, 2014). Sexes were analyzed separately and *P*‐values were obtained from *F*‐statistics.

### Larval transcriptome sequencing

Larval samples were ground to fine powder in 1.5 mL tubes placed in liquid nitrogen. Samples were mixed with 500 μL of lysis buffer (containing 1% *β*‐mercaptoethanol) from Nucleospin® RNA extraction kits (Macherey‐Nagel GmbH & Co. KG, Düren, Germany) and vortexed to complete homogenization. RNA extraction and purification were performed using Nucleospin® RNA columns (Macherey‐Nagel) according to manufacturer's instructions. Total RNA was eluted in 50 μL of RNase‐free water. RNA was quantified and quality‐checked using the NanoDrop ND‐1000 spectrophotometer (NanoDrop Technologies, USA) and by running 200 ng of total RNA on 1% agarose gel. Quality control was also performed by Agilent Bioanalyzer Picochip (Agilent, Palo Alto, CA) which ensured high quality of all extractions (Fig. S1). Illumina TruSeq stranded mRNA‐Seq libraries were prepared and sequenced on an Illumina HiSeq 2500 instrument (Eurofins Genomics, Ebersberg, Germany), generating 100 bp paired‐end reads.

The quality of the raw reads was examined in FASTQC 0.11 (Andrews 2010) and MULTIQC 1.7 (Ewels *et al.*, 2016). Bases with a phred score below 20 and Illumina adapter read‐through of at least 3 bp were trimmed from the 3’ end using TRIMGALORE 0.6.4 (Krueger, 2015). Trimmed reads were aligned to the *D. melanogaster* release 6 genome (GCF_000001215.4) using HISAT 2.1.0 (Kim *et al.*, 2019) and alignments were processed using SAMTOOLS 1.9 (Li *et al.*, 2009). To estimate the fraction of males in each larval pool, we obtained read coverage per chromosome using QUALIMAP 2.2.1 (Okonechnikov *et al.*, 2016), standardized coverage to exome size as obtained from exon annotations using BEDTOOLS 2.28 (Quinlan & Hall, 2010) and calculated relative Y‐chromosome coverage [Y/(X + Y)], expected to range from 0 (no males) to 0.5 (all males) (Palmer *et al.*, 2019).

### Differential gene expression

Read alignments were quantified against exon annotations and summarized at the gene level using FEATURECOUNTS 1.6.2 (Liao *et al.*, 2014), considering only concordantly mapped read pairs and assigning multimappers as fractional counts to all mapping locations to avoid multicopy gene families dropping out of the analysis. Differential gene expression analysis among the three treatment groups was carried out with *DESeq2* 1.26.0 (Love *et al.*, 2014) in R 3.6.1 (R Core Team, 2019), shrinking fold‐changes of low‐count genes using the *apeglm* method (Zhu *et al.*, 2019). To account for confounding differences in sex‐specific trade‐off responses (Magwere *et al.*, 2004) due to varying sex‐ratios among pools, the model incorporated relative Y‐chromosome coverage as a covariate. Differentially expressed genes (DEGs) were selected at an FDR‐corrected (Benjamini & Hochberg 1995) *P*‐value cut‐off (*q*‐value) of 0.05 and an absolute fold‐change cut‐off of at least 1.5×. Overlaps of DEG lists between the three contrasts were inspected using intersection plots generated using *UpsetR* 1.4.0 (Conway *et al.*, 2017). Expression heatmaps were constructed using *gplots* in R (Warnes *et al.*, 2016). To validate RNAseq data, expression levels of 20 candidate DEGs were assessed with RT‐qPCRs using the same (reverse‐transcribed) RNA extracts (Table S1).

### KEGG pathway and GeneOntology annotation and enrichment

We assigned KEGG pathway annotations to each gene using the KEGG pathway database (https://www.genome.jp/kegg/pathway.html) API as implemented in CLUSTERPROFILER 3.14.3 (Yu *et al.*, 2012). Gene names were converted to NCBI ENTREZ identifiers using the Bioconductor org.Dm.eg.db_3.10.0 database. We first examined the fold‐change distributions of genes within each of the five main KEGG classes (Cellular Processes, Environmental Information Processing, Genetic Information Processing, Metabolism and Organismal Systems) and compared medians using the Kruskal–Wallis test. Second, we calculated the root‐sum‐square of fold‐changes within each KEGG class (regulation score). Third, we tested for significant functional overrepresentation of individual KEGG pathways against the *D. melanogaster* whole‐genome background among sets of DEGs common across or idiosyncratic to specific density contrasts. Significantly overrepresented KEGG pathways were selected at an FDR‐corrected *P*‐value cut‐off (*q*‐value) of 0.05. Finally, we constructed a network illustrating the overlap in genes between the significantly enriched KEGG pathways in Cytoscape version 3.7.1 with the plugin EnrichmentMap (default analysis type setting) (Shannon *et al.*, 2003; Merico *et al.*, 2010). Overrepresentation of GeneOntology (GO) terms in the *biological process* ontology was examined using CLUSTERPROFILER, keeping terms at GO levels 1–4 and clustering terms by semantic similarity based on a Wang distance cutoff of 0.5 (Wang *et al.*, 2007) and retaining the term with the lowest *P*‐value in each cluster.

## Results

### Larval density effects on adult phenotypes

**Fig. 1:**
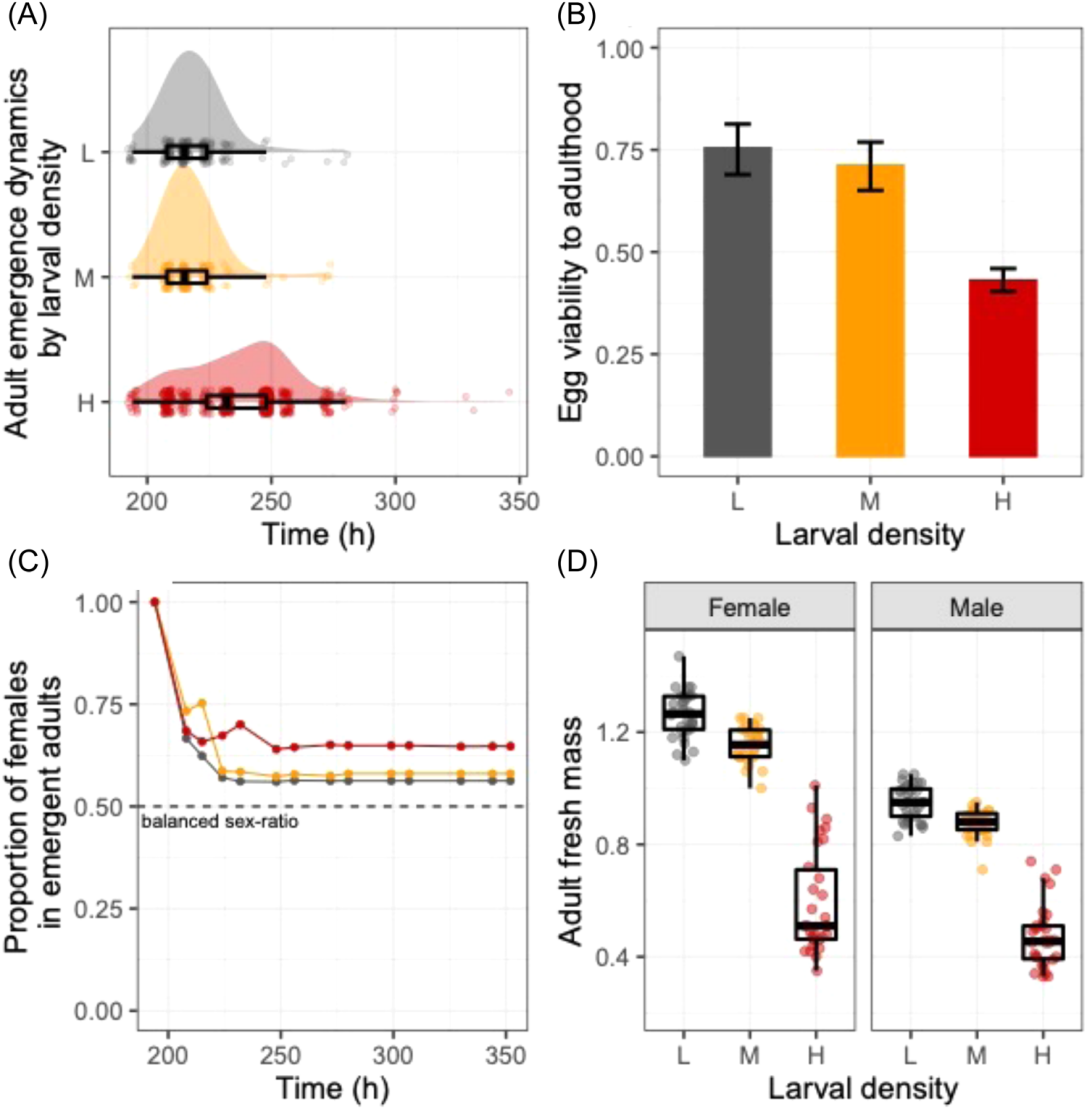
Effects of larval density on adult phenotypes. (A) Adult emergence dynamics time. (B) Egg to adult viability. (C) Proportion of females in emergent adults. (D) Body mass. Three larval densities were tested: L (low density; 5 eggs/mL; in gray), M (medium density; 60 eggs/mL; in orange) and H (high density; 300 eggs/mL; in red). Error bars in B are 95% confidence intervals.

We subjected *D. melanogaster* larvae to three density treatments (low density; 5 eggs/mL), M (medium density; 60 eggs/mL), and H (high density; 300 eggs/mL) and examined whether developmental time, adult body weight, and sex ratio at emergence differed between treatments. Larval density affected the development dynamics, with individuals from the H treatment taking longer to reach adulthood than those from L and M (*t*‐test, both adjusted *P* < 0.001) (Fig. 1A). Viability from egg to adult was also impaired at higher densities, dropping from 76% (CI95: 69–88) in L to 73% (CI95: 67–76) in M and to 46% (CI95: 43–48) in H (Fig. 1B). The sex‐ratio of adult flies was slightly skewed toward females in all densities, ranging from 56% in L to 64% in H (Fig. 1C). Larval density significantly affected both females’ (*F*_2,87_ = 261.54; *P* < 0.001) and males’ (*F*_2,87_ = 319.18; *P* < 0.001) dry body mass, whereby the H treatment generated adults that were significantly lighter than adults from L or M treatments (Fig. 1D). These observations confirm that the H treatment was a resource‐limiting and potentially stressful developmental environment for individuals, while the medium‐density treatment had only minor effects.

### Larval density effects on larval transcriptional patterns

**Fig. 2:**
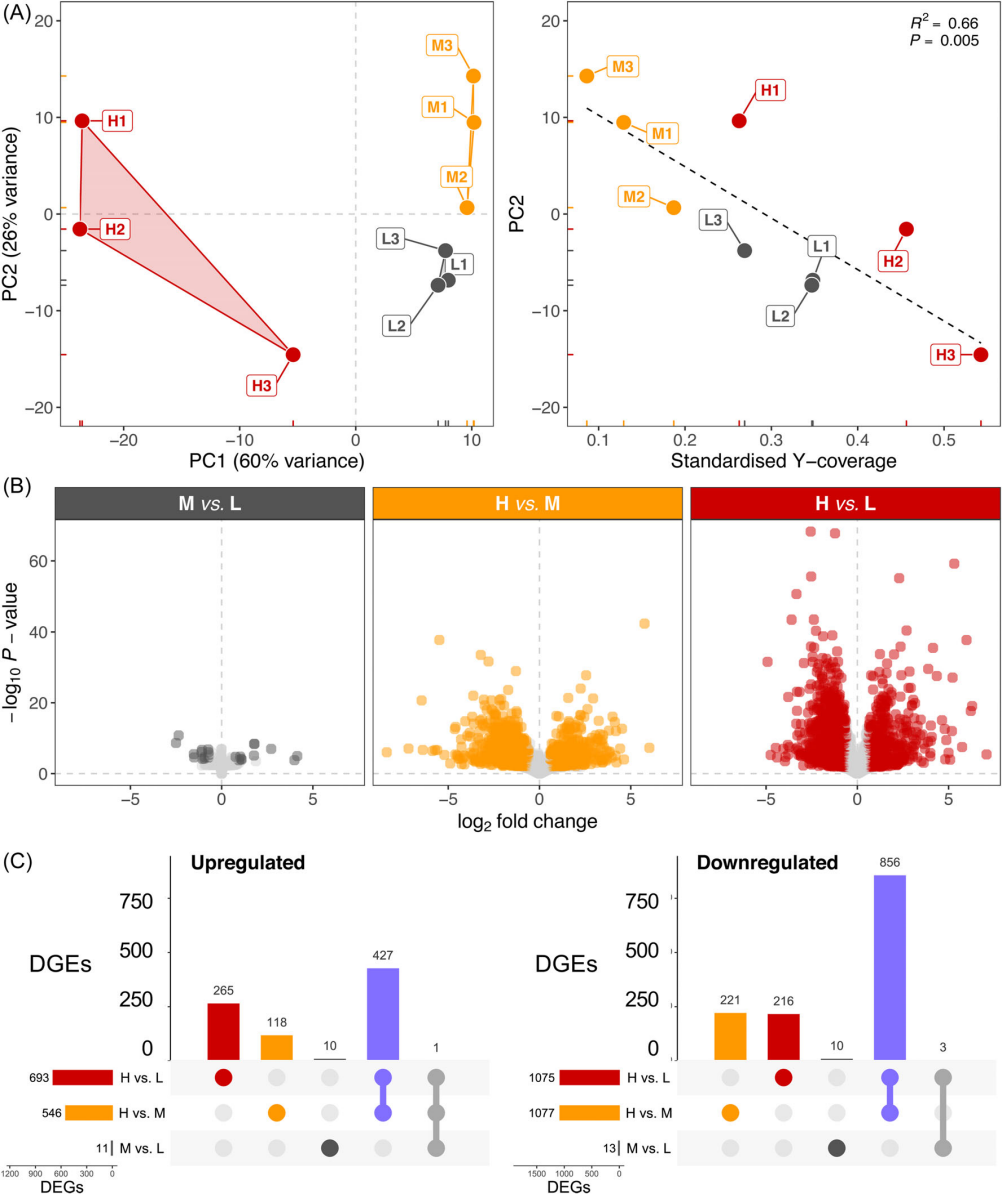
Effects of larval density on larval gene. (A) Left: Scatter plot of the first two principal components (PC1/PC2) of variance‐stabilized normalized read counts among samples Right: Scatter plot and linear regression of PC2 against standardized Y‐coverage (proxy for sex ratio in larval pool). The coefficient of determination (*R* ^2^) and the *P*‐value of the linear model are displayed on the top right. Samples are labeled and colored by density treatment as in Fig. 1. (B) Volcano plots summarizing expression change (log_2_ fold‐change) and statistical significance (−log_10_ *P*‐value) for each gene in medium/low (M vs. L), high/medium (H vs. M) and high/low (H vs. L) larval density contrasts. Significant DEGs (FDR ≤ 0.05, fold‐change ≥ 1.5×) are highlighted in dark gray (M vs. L), amber (H vs. M), or red (H vs. L). (C) Intersection plots summarizing overlap between upregulated (left) or downregulated (right) DEGs among the three contrasts. Four key intersections are highlighted in dark gray (M vs. L only), amber (H vs. M only), red (H vs. L only) and purple (DEGs common between H vs. M and H vs. L contrasts).

Having confirmed our phenotypic effects of our larval manipulation, we then investigated the associated transcriptional responses to larval crowding in three larval pools per treatment. We generated 18 988 311 to 28 154 203 high‐quality read pairs per pool, of which 97.4%–97.8% aligned to the genome and 82.3%–86.9% of alignments (including 6.9% to 11.5% from multimapping reads) were quantified against gene annotations (Table S2). The reads covered 91.3% of the 17 759 annotated reference genes; ignoring multimapping reads decreased this proportion to 90.9% and had no effect on the interpretation of the results. To estimate sex ratio in each pool, we identified relative Y‐chromosome coverage, which ranged from 0.09 to 0.54 (suggesting 2–10 males per pool; Table S2) and varied among density groups (*F*_2,6_ = 7.57; *P* = 0.02). Principal component analysis (PCA) on the variance‐stabilized normalized read counts showed clear separation between the three larval density groups on the first axis (60% of variance explained; Fig. 2A). The second PCA axis explained 26% of the variance and was significantly associated with standardized Y‐coverage (*F*_1,7_ = 16.42; *P* = 0.005; adjusted *R* ^2^ = 0.66; Fig. 2A). The lowest Y‐coverage was observed at medium density, whereas samples at high density had highest Y‐coverage but also large variation, overlapping with samples from low density (Fig. 2A).

To account for this sex‐specific effect, we incorporated standardized Y‐coverage as a numeric covariate into the DGE model. The transcriptomic response was entirely consistent with phenotypic effects, whereby medium density had a weak effect and high density had a large effect on gene expression (Fig. 2B). Fold‐changes within the three contrasts were tightly correlated with qPCR‐based fold‐changes (*R* ^2^ ranging from 0.95 to 0.97; Table S1). Only 24 differentially expressed genes (DEGs) were observed at medium density (M vs. L contrast), whereas 1623 and 1768 DEGs were observed at high density (H vs. M and H vs. L contrasts, respectively) (Fig. S2, Table S3). The union of DEGs among these two high‐density contrasts comprised 2107 DEGs, of which 1284 DEGs were shared, representing the “core” high‐density crowding response (Fig. S2). Considering overlaps among up‐ and downregulated DEGs separately among all contrasts resulted in a core crowding response of 427 up‐ and 856 downregulated genes (Fig. 2C, Table S3). For example, the *Hr38*, *ng1*, and *ng3* genes were among the most strongly upregulated genes in the core crowding response (Table S3), and *ng2* was the only upregulated gene common across all three contrasts (Fig. 2C). The H vs. M and H vs. L contrasts also contained 118 or 265 upregulated and 221 or 216 downregulated unshared (“idiosyncratic”) DEGs, respectively (Fig. 2C, Table S3). Although 21 of 24 DEGs at medium density were shared with other contrasts (Fig. S2), the direction of regulation was opposite from that at high density for 20 of 24 DEGs (Fig. 2C, Table S3). For example, *phu* was the most strongly upregulated gene at medium density but was among the most strongly downregulated genes at high density (Table S3).

### Biological pathway analysis of differentially expressed gene sets

**Fig. 3:**
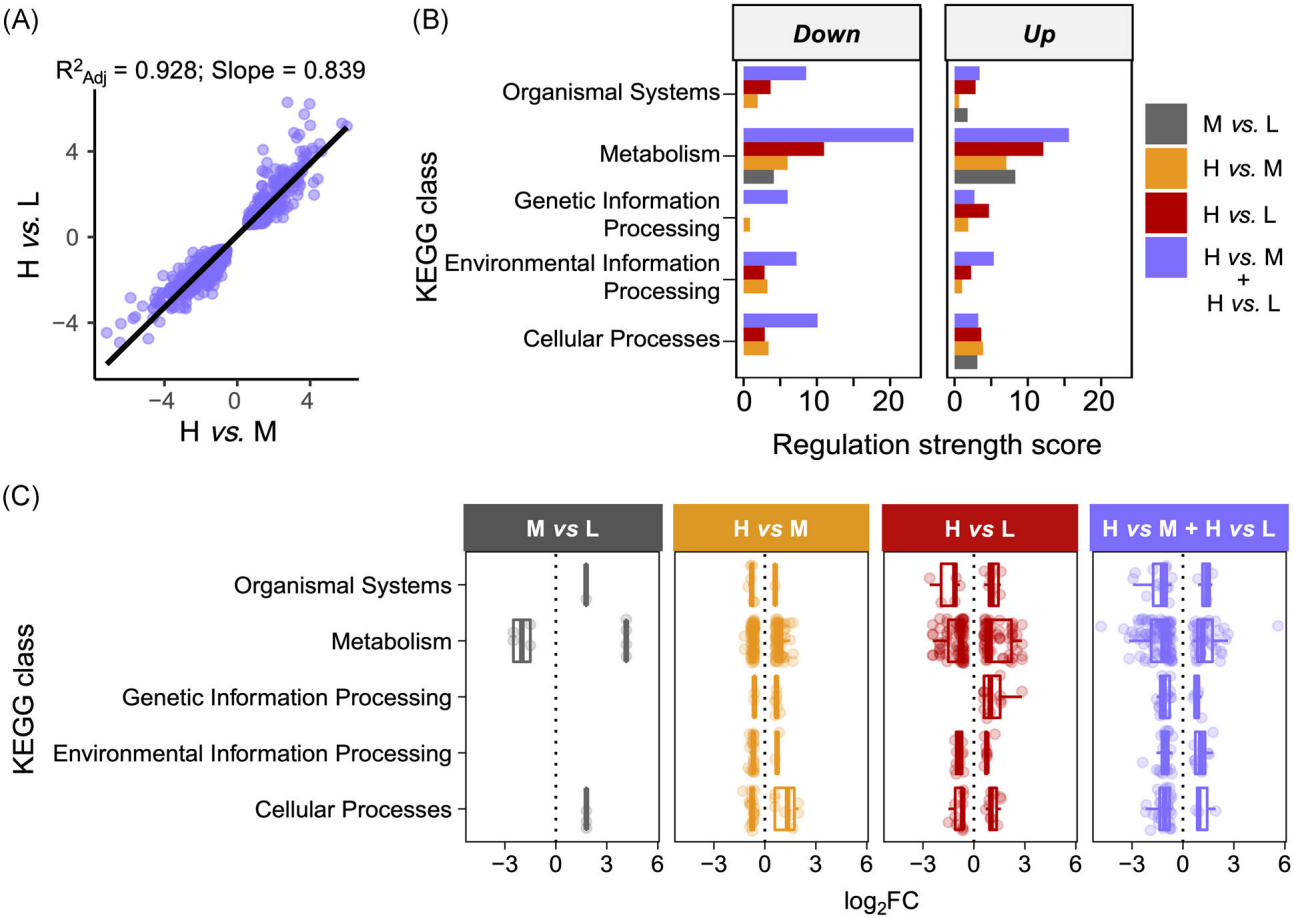
KEGG pathway regulation among DEGs responding to larval crowding. (A) Correlation of log_2_ fold‐changes of DEGs in core crowding response between the two high‐density contrasts (H vs. M and H vs. L). (B) Regulation strength scores (root‐sum‐square of fold‐changes) for each KEGG class based on the DEGs for the core crowding response and for idiosyncratic DEGs in M vs. L, H vs. M, and H vs. L contrasts. (C) Genes log_2_FC organized according to KEGG class with boxplots superimposed for up‐ and downregulated genes separately. Each dot represents a gene and dots are colored according to contrast.

Next, we examined biological pathway annotations in each identified shared and idiosyncratic (unique) gene set among density contrasts since these may represent distinct ecophysiological processes. The core crowding response (i.e., purple intersection in Fig. 2C) comprised 1273 genes with strongly correlated fold‐changes between the H vs. M and H vs. L contrasts (*R* ^2^ = 0.928; Figs. 3A and S2). Of these, 944 (75%) and 265 genes (21%) were annotated with GeneOntology (GO) terms from the *biological process* ontology and KEGG pathways, respectively (background annotation rates: 76% and 20%). Most of the KEGG annotations were from the “Metabolism” class, which displayed by far the largest root‐sum‐square fold‐change among all five KEGG classes, for both up‐ and downregulated DEGs (Fig. 3B). This strong regulation of metabolism was also observed for the idiosyncratic components of all three density contrasts (Fig. 3C).

**Fig. 4:**
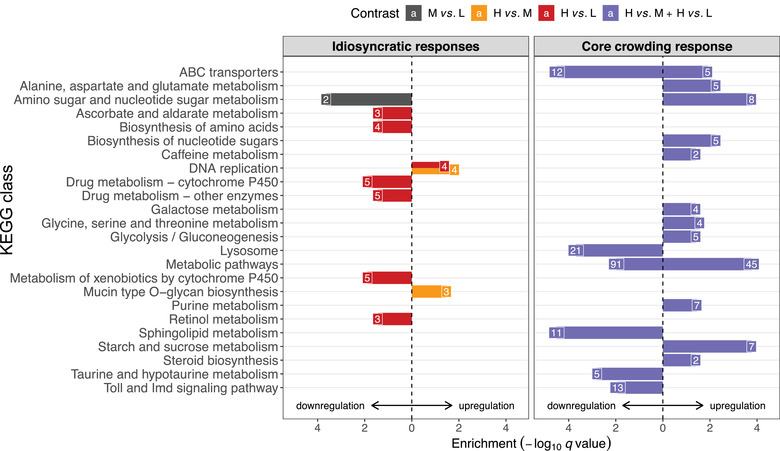
KEGG pathway enrichment in DEGs unique to individual density contrasts (idiosyncratic responses) or common in high/low and high/medium contrasts (core crowding response). The statistical significance (−log_10_ *q* value) is plotted for each KEGG pathway (*q* ≤ 0.05), whereby the direction of the bar indicates up‐ or downregulation of the underlying DEGs. The numbers of DEGs annotated with the enriched pathway are indicated at the end of each bar. Four key intersections are highlighted in dark gray (M vs. L only), amber (H vs. M only), red (H vs. L only), and purple (DEGs common between H vs. M and H vs. L contrasts).

**Fig. 5:**
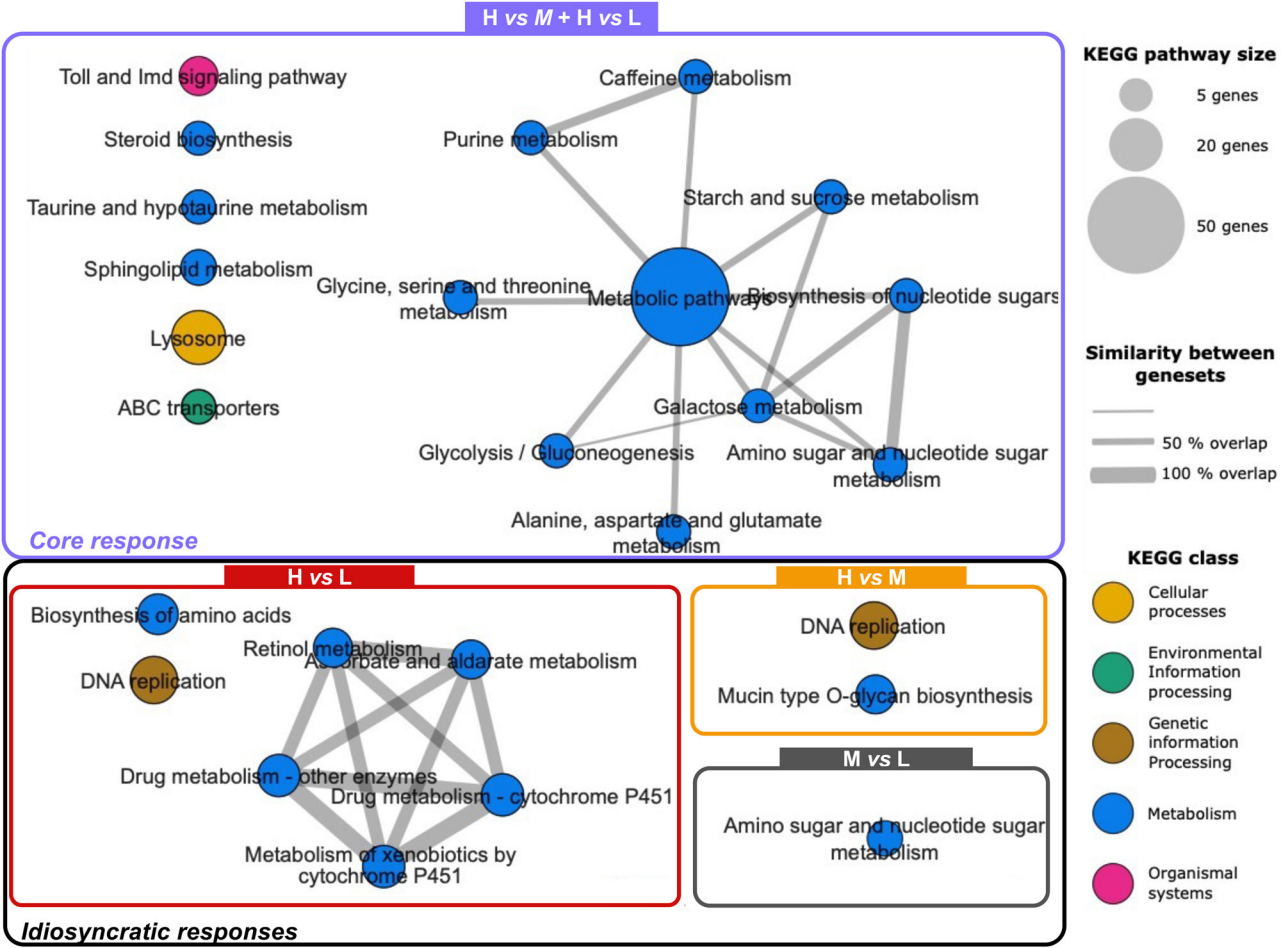
KEGG pathway similarity network for DEGs unique to three individual density contrasts (idiosyncratic responses) or common in H vs. M and H vs. L contrasts (core crowding response). The nodes represent the KEGG pathways significant at each comparison and the edges represent the similarity between the different KEGG pathways (i.e., overlap in genes). The size of the nodes represents the number of genes in the pathway with bigger nodes representing more genes. The width of the edges between the nodes represents the similarity coefficient. The thicker the edge the more genes they have in common. Pathways are colored according to KEGG classification.

We then performed KEGG pathway enrichment (overrepresentation) analysis on the core crowding response and idiosyncratic responses. The upregulated DEGs in the core crowding response supported overrepresentation (FDR ≤ 0.05) of eleven pathways, including several metabolism pathways, purine metabolism, and steroid biosynthesis (Fig. 4, Table S4). The downregulated DEGs supported overrepresentation of sphingolipid metabolism, lysosome, taurine metabolism, and Toll and Imd signaling pathways (Fig. 4, Table S4). The *Metabolic pathways* and *ABC transporters* pathways were significantly overrepresented both among up‐ and downregulated DEGs (Fig. 4, Table S4). There was little overlap in genes among these pathways, suggesting that these represent independent physiological responses (Fig. 5). The idiosyncratic response to medium crowding (M vs. L) indicated significant overrepresentation of the *Amino sugar and nucleotide sugar metabolism* pathways among downregulated DEGs, but no significant overrepresentation of any pathway among upregulated DEGs (Fig. 4, Table S4). The two idiosyncratic responses to high crowding in the H vs. M and H vs. L contrasts indicated significant overrepresentation of *DNA replication* (both H vs. M and H vs. L) and *Mucin type O‐glycan biosynthesis* (H vs. M. only) among upregulated DEGs, as well as multiple *P450 xenobiotics metabolism pathways* (H vs. L only) among downregulated DEGs (Fig 4, Table S4). Due to gene sharing with the P450 pathways, retinol metabolism and ascorbate and aldarate metabolism were also overrepresented among downregulated DEGs (Fig. 5).

Overrepresentation tests of GeneOntology terms in the *biological process* ontology yielded similar results to the KEGG analysis, highlighting upregulation of energy metabolism and downregulation of homeostasis and immune defense in the core crowding response, as well as downregulation of drug metabolism and developmental growth in idiosyncratic responses (Fig. S3).

## Discussion

We present the first transcriptome‐wide overview of responses to larval crowding in *Drosophila melanogaster*. Our results show that larval transcriptomic responses are consistent with the known negative adult phenotypic effects of larval overcrowding. While medium crowding had little effect on adult phenotypes and larval gene expression, high crowding had substantial phenotypic and transcriptomic effects, which were distinct from those at medium density. These data allow us for the first time to test predictions from previous targeted studies and provide novel candidate genes and physiological pathways for studying the ecological processes affected by larval overcrowding.

We predicted that high crowding can lead to accumulation of toxic compounds, which necessitates molecular responses to cope with a toxic developmental environment (Table 1). We found that high crowding caused marked transcriptional changes in metabolic pathways and pathways related to growth and toxicity. This is consistent with decreased protein availability in the larval diet and an accumulation of toxic waste compounds, with knock‐on effects for larval growth (Henry *et al.*, 2018; Klepsatel *et al.*, 2018; Henry *et al.*, 2020). For instance, we found that steroid metabolism was upregulated, including lipases *Lip3* and *Lip4*. This is in agreement with recent findings showing that *Lip3* is upregulated under responses to extreme dietary stress (starvation) (Hänschke *et al.*, 2022). Interestingly Klepsatel *et al.* (2018) reported that flies that had developed under high larval density had reduced protein content and were fatter than the flies from standard density. Likewise, Zwaan *et al.* (1991) reported that the fat content of flies increased with increasing crowding. Hence it appears that crowding triggers a shift in metabolic pathways leading to triglyceride (fat) accumulation. This may be related to limiting dietary yeast/proteins in diet under crowded conditions, whereby the ratio of protein:carbohydrate decreases rapidly in crowded larval conditions resulting in increased responses to metabolic responses to carbohydrate (i.e., Skorupa *et al.*, 2008; Klepsatel *et al.*, 2018). Molecular responses to protein:carbohydrate ratios have been observed in *Drosophila* adult and therefore, are plausible in larvae (Post & Tatar, 2016). Likewise, a metabolic remodeling hypothesis was also suggested by metabolomic profiling data which revealed dissimilar metabotypes among larval densities, which were mainly characterized by high concentrations of sugars and low amounts of amino acids at higher densities (Henry *et al.*, 2018). We also found that glycolysis, gluconeogenesis and galactose metabolism were upregulated probably as a by‐product of elevated food uptake due to compensatory feeding to satisfy for protein requirements (Skorupa *et al.*, 2008; Klepsatel *et al.*, 2018). *Hr38* was among the most strongly upregulated genes, consistent with its role in regulating carbohydrate metabolism and glycogen storage of growing larval stage (Ruaud *et al.*, 2011). Moreover, purine metabolism was also upregulated as shown by the *uro* gene, which corroborates previous findings showing that *uro* is a key gene in the response to larval crowding (Henry *et al.*, 2018). Sphingolipid and taurine metabolism as well as lysosome and ABC transporters were downregulated suggesting that excretion and recycling pathways may be negatively impacted in crowded conditions. Together, these results show that larval crowding modulates the response to low nutrition and high toxicity.

High crowding also caused strong downregulation of immunity via the Toll/Imd pathways involving two PGRPs (*PGRP‐SD* and *PGRP‐LA*) and *spätzle*, which resulted in downregulation of antimicrobial peptide expression including *DptA*, *Drs*, and *Drsl5*. This finding challenges the prediction that *Drosophila* larvae should upregulate immune function due to density‐dependent prophylaxis as in other insects (e.g., Cotter *et al.*, 2004; Siva‐Jothy *et al.*, 2005). However, it is possible that the larval densities used here are above the larval densities which stimulate immunity. Nevertheless, the repression of transcripts involved in immunity found here is likely responsible for increased larval mortality in crowded conditions found here and elsewhere (Than *et al.*, 2020), which imposes a strong selective pressure for populations experiencing high larval densities. In *Drosophila*, populations adapted to larval overcrowding evolve pathogen‐specific responses against Gram‐positive pathogens, suggesting that larval crowding might increase the risk of immune challenges by Gram‐positive pathogens in the population (Kapila *et al.*, 2021). Crowding directly alters the nutritional properties of the diet, not only quantitatively but also qualitatively, with accumulation of metabolic wastes and decaying dead carcasses (Henry *et al.*, 2018; Henry *et al.*, 2020). This in turn triggers marked changes in the bacterial composition of food with, for instance, the appearance of genus like *Pseudomonas* (Henry *et al.*, 2020). These bacteria are well known for their pathogenicity toward *Drosophila* (D'Argenio *et al.*, 2001). Hence, the emergence of pathogens in food concurrently with a reduced immunity may be, at least in part, a driver of lethality at high larval density.

We also hypothesized that autophagy pathways would be upregulated in the core responses to crowding due to the reported increased lifespan in adults from crowded larval environments (Shenoi *et al.*, 2016; Klepsatel *et al.*, 2018) (Table 1). This is because disruption of autophagy is a known factor in aging (Maruzs *et al.*, 2019). Our results do not directly support our predictions as our KEGG analysis did not detect an enrichment of the autophagy pathway. However, our analysis showed that the lysosome pathway, which is indirectly related to autophagy as well as directly involved in other biological processes, is downregulated in the core responses to high crowding. For instance, *LERP* is a receptor that is important lysosome vesicle trafficking as well as for eye development related to autophagy (Hasanagic *et al.*, 2015). *LERP* knockdown results in lower body bass and increased starvation sensitivity in adult flies, which are traits associated with development in crowded environments (Than *et al.*, 2020). Similarly, we found that *Npc2a* was downregulated, which is an important gene regulating the sterol metabolism and molting in *Drosophila* larvae (Huang *et al.*, 2007). *Ppt2* was also downregulated and is a gene linked to neuronal development in *Drosophila* (O'Hern 2011). *Ppt2* knockdown showed abnormal neuronal development which could lead to impaired functions. This can help explain why *Drosophila* larvae developing in crowded conditions showed impaired learned visual recognition (Slepian *et al.*, 2015). Autophagy is not only used for recycling damaged organelles or proteins; it also provides nutrients to maintain important cellular functions. Lipids including sphingolipids are increasingly recognized as key regulators of a series of critical cellular processes such as autophagy (Hebbar *et al.*, 2015). We found here that genes involved in sphingolipid metabolism were also downregulated in the core responses to larval crowding. Studies in flies have underscored the roles of sphingolipids in controlling lipid storage and response to nutrient availability (Kraut, 2011). For instance, changes in sphingolipid metabolic genes are part of the transcriptional response to sugar feeding of *Drosophila* larvae (Zinke *et al.*, 2002). This study identified an acid sphingomyelinase (aSMase) and a ceramidase (CDase) both as being downregulated after sugar feeding, whereas fatty acid synthesis was upregulated. We also found that CDase was downregulated in response to crowding, as well as *schlank*, a ceramide synthase, and the ceramide kinase (*Cerk*), which catalyzes phosphorylation of ceramide. Taken together, our results highlight sphingolipid metabolism is important during crowding, either for the regulation of cell death (autophagy) or for the metabolic regulation of nutrient scarcity.

Larval crowding was expected to trigger the expression of heat‐shock proteins in a hormesis‐like response in our experiment (Henry *et al.*, 2018; Lushchak *et al.*, 2019). However, our data did not show increased expression of heat‐shock proteins at high density, suggesting that hormesis‐like responses may not be as strong as previously considered under the circumstances tested in this study. In a target gene expression study, Henry *et al.* (2018) also failed to find strong upregulation of *hsp* family genes in M and H larvae, corroborating the present results. Thus, it appears that effects of crowding (beneficial or detrimental) are unrelated to heat‐shock response. Nevertheless, high crowding downregulated the autophagy and lysosome pathways (i.e., recycling pathways), which could lead to a generalized stress response if damages in protein and other cellular components accumulate in individuals experiencing larval crowding. This accumulation of stress could underpin hormesis‐like responses in adulthood. It is possible that our design, which sampled wandering larvae, selectively excluded larvae that accumulated higher stress levels as those were likely slower to pupate. This would explain the contradictory results presented here and the literature. Alternatively, it is possible that specific genes of the stress pathways are upregulated but not enough to be detected in our differential expression analysis. Future studies that directly manipulate larval stress levels will provide greater insights into the genes as well as overall transcriptional responses involved in organismal responses to stress and will allow us to compare how those pathways are affected by larval crowding.

Larval crowding is also expected to affect reproductive processes, for example by increasing testis sizes relative to body mass (Johnson *et al.*, 2017; Than *et al.*, 2020; Morimoto *et al.*, 2021). Males and females developing in crowded larval environments are smaller and with lower mating and reproductive success, displaying higher investment in mating opportunities (Wigby *et al.*, 2016; Than *et al.*, 2020). Moreover, phenotypic effects caused by larval crowding can persist through to next generations (Morimoto *et al.*, 2017). While our phenotypic data is consistent with these ideas, the transcriptomic data had no evidence of direct changes in reproductive pathways. One important limitation of our experimental design was that our larval pools had unknown sex‐ratios and thus did not allow to specifically test for sex‐specific effects on transcription. However, our sex‐ratio estimates based on Y‐chromosome coverage suggested that our pools differed in sex‐ratio and that the transcriptomic response is affected by variation in sex‐ratio. This is consistent with previous reports of sex‐specific responses in reproduction to larval crowding (Baldal *et al.*, 2005; Vishalakshi & Singh, 2008; Kapila *et al.*, 2021) and warrants future efforts into ascertaining sex‐specific crowding‐associated trade‐offs. It is important to mention that Y‐chromosome coverage was highest in the high‐density group, which suggests a male‐biased sex‐ratio, but the data for adult emergence suggest a female‐biased treatment. This is because for the former, Y‐chromosome coverage was estimated from RNAseq of the subset of wandering larvae collected for the experiment (see Materials and methods) whereas the emergence data takes into account the total number of individuals that emerged. The former is naturally affected by stochasticity during sampling (particularly for larvae, which does not present morphological sex dimorphism). This sampling stochasticity was present across all treatments in a similar fashion.

Three out of the four genes most strongly upregulated in response to high crowding were new glue genes *ng1*, *ng2*, *ng3*. These genes are normally transcribed from the beginning of the third instar and their expression is repressed later to allow the puffing stages transition to proceed in salivary gland chromosomes (D'Avino *et al.*, 1995). These intermolt puffs are regulated by ecdysone before the onset of metamorphosis, whereby an ecdysone peak can trigger key developmental events such as the cessation of feeding and the initiation of wandering of the larvae (Dominick & Truman, 1985). In *D. melanogaster* this ecdysone peak is also responsible for the repression of *ng1*, *ng2*, and *ng3* (Furia *et al.*, 1990; D'Avino *et al.*, 1995). In this study, we only sampled wandering larvae and the relative high expression of *ng* genes in crowded condition suggests a possible disturbance of ecdysone‐mediated developmental processes (e.g., longer puffing stage transitions). This would be consistent with the observation that larval crowding affects the development dynamics, with individuals at high larval density taking longer to develop to adulthood than those from medium or low density.

The majority of the effects in molecular pathways belonged to the core responses to crowding, whereas the idiosyncratic responses to crowding revealed a relatively small number of pathways. For instance, medium crowding resulted in a small decrease in body mass and downregulation of *Cht4* and *Cht9* genes involved in the amino sugar and nucleotide sugar metabolism pathway. A further increase in larval crowding resulted in strong phenotypic effects as shown by a reduction in egg‐to‐adult viability and adult body weight (Fig. 1), but upregulation of only repair enzymes of the DNA replication pathways (*Mcm* gens) and *Pgant* genes involved in mucin‐type O‐glycan biosynthesis pathways (H vs. M). Similar patterns were observed in the comparison between L and H, where the DNA replication pathway (*Mcm* and *DNApol* genes) were upregulated. Moreover, we also found that genes related to the metabolism of toxic compounds, including *Ugt* and *Gst*, were downregulated. In insects*, Ugt* genes are known to modulate the conjugation of glucose to eliminate toxic compounds and play a role in several physiological processes, including insecticide resistance (Lee *et al.*, 2005; Ahn & Marygold, 2021). Likewise, *Gst* are also involved in detoxification and insecticide resistance (Le Goff *et al.*, 2006; Willoughby *et al.*, 2006). Therefore, H larvae have different susceptibility to toxic compounds relative to L larvae. Thus, increasing larval crowding has a consistent effect on molecular pathways above and beyond the magnitude of phenotypic effects (i.e., core response), and only specific pathways are regulated on a density‐specific manner (i.e., idiosyncratic responses). Other Omics results also suggest that larval crowding generated physiological phenotypes that are density‐specific (Henry *et al.*, 2018). It is important to highlight that the *phurba tashi* (*phu*) gene, which is involved in hypoxia responses in *Drosophila* (Mossman *et al.*, 2017), showed a density‐dependent expression pattern, whereby *phu* was strongly upregulated in M larvae but amongst the most downregulated in the H larvae. These density‐specific physiological and molecular signatures may underlie hormetic responses triggered by crowding (Henry *et al.*, 2018; Lushchak *et al.*, 2019). It is also worth mentioning that we sampled wandering larvae, which is a dynamic stage for transcriptional changes. While we standardized the sampling to account for asynchronies, it is still possible that some of the idiosyncratic responses observed here emerge from small (but random) effects of sampling time. Future studies standardizing sampling relative to Zeitgeber time may minimize these potential effects.

Overall, we show that larval crowding induces changes in the transcriptomic profile of *Drosophila melanogaster* larvae. These responses are primarily related to metabolic pathways, but also include pathways related to growth and maintenance and toxicity. In particular, sugar, steroid and amino acid metabolism pathways as well as DNA replication, ABC transporters, taurine metabolism, P450 xenobiotics metabolism and the Toll/Imd signaling pathways were identified as key pathways underpinning the molecular responses to larval overcrowding and thus, are candidates for future targeted studies. For instance, it will be important to characterize the specific role of the differentially expressed genes and pathways on the response to larval crowding across holometabolous insects, allowing for a comparative understanding of the molecular responses to intraspecific competition. Importantly, the molecular insights gained here help answer disparate questions in the field of developmental ecology, by shedding light into how molecular pathways respond to a stressful and competitive developmental environment. Overall, our study shows the molecular pathways underpinning larval responses to developmental environments and broadens our understanding of how holometabolous insects respond to intraspecific competition during development.

## Data Archiving

Raw sequence data and the processed gene‐count matrix have been deposited at the NCBI Gene Expression Omnibus database (Edgar *et al.*, 2002) and are accessible through GEO Series accession number GSE193120.

## Disclosure

All authors have seen and agree with the contents of the manuscript and there is no conflict of interest, including specific financial interest and relationships and affiliations relevant to the subject of manuscript.

## Supporting information


**Fig. S1** RNA electropherograms from Eukaryote Total RNA Pico assay.
**Fig. S2** Overlaps of differentially expressed genes (DEGs) in three density contrasts.
**Fig. S3** GeneOntology (biological process) enrichment in DEGs unique to individual density contrasts (idiosyncratic responses) or common in high/low and high/medium contrasts (core crowding response).

**Table S1** Methods and Results of qPCR experiments to corroborate RNAseq data (separate Excel).

**Table S2** RNA‐Seq QC and alignment statistics (separate Excel).

**Table S3** Full DGE results and intersections (separate Excel).

**Table S4** KEGG enrichment results (separate Excel).
