# Supplementary material for: The transcriptomic signature of responses to larval crowding in Drosophila melanogaster
Source: Insect Sci. 2022 Oct 17;30(2):539–54. doi: 10.1111/1744-7917.13113 (PMC10947363; doi:10.1111/1744-7917.13113)
Supplement: Supplementary file 1 — Fig. S1 RNA electropherograms from Eukaryote Total RNA Pico assay. Fig. S2 Overlaps of differentially expressed genes (DEGs) in three density contrasts. Fig. S3 GeneOntology (biological process) enrichment in DEGs unique to individual density contrasts (idiosyncratic responses) or common in high/low and high/medium contrasts (core crowding response). [file INS-30-539-s004.docx]

**Supplementary Material: “The transcriptomic signature of physiological trade-offs caused by larval overcrowding in *Drosophila melanogaster*”**

Juliano Morimoto^1,2,3^*, Marius Wenzel^1^, Davina Derous^1^, Youn Henry^4, 5^, Herve Colinet^4*^

1. School of Biological Sciences, University of Aberdeen, Tillydrone Avenue, AB24 2TZ, United Kingdom
2. Programa de Pós-graduação em Ecologia e Conservação, Universidade Federal do Paraná, Curitiba, 82590-300, Brazil
3. Institute of Mathematics, University of Aberdeen, King's College, Aberdeen AB24 3FX
4. University of Rennes, CNRS, ECOBIO [(Ecosystèmes, biodiversité, évolution)] - UMR 6553, F 35000 Rennes, France
5. Department of Ecology and Evolution, University of Lausanne, CH 1015 Lausanne, Switzerland

*Correspondence

[juliano.morimoto@abdn.ac.uk](mailto:juliano.morimoto@abdn.ac.uk)

[herve.colinet@univ-rennes1.fr](mailto:herve.colinet@univ-rennes1.fr)

# Supplementary Tables

**Table S1.** Methods and Results of qPCR experiments to corroborate RNAseq data (separate excel).

**Table S2.** RNA-Seq QC and alignment statistics (separate excel).

**Table S3.** Full DGE results and intersections (separate excel).

**Table S4.** KEGG enrichment results (separate excel).

# Supplementary Figures

**Figure S1.** RNA electropherograms from Eukaryote Total RNA Pico assay

**
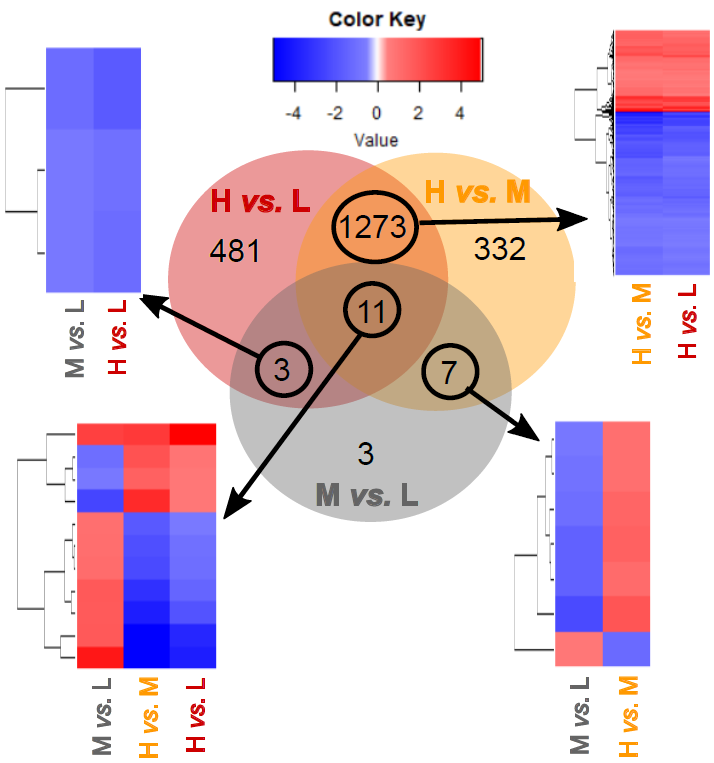
**

**Figure S2. Overlaps of differentially expressed genes (DEGs) in three density contrasts**. Patterns of gene expression in each intersection are visualised as heatmaps of log_2_ fold change (blue=downregulation; red=upregulation). M *vs.* L, H *vs.* M and H *vs.* L refers to medium-density versus low-density, high-density versus medium-density and high-density versus low-density respectively.

**Figure S3.** **GeneOntology (biological process) enrichment in DEGs unique to individual density contrasts (idiosyncratic responses) or common in high/low and high/medium contrasts (core crowding response).** The statistical significance (-log_10_ *q* value) is plotted for each term (*q* <= 0.05), whereby the direction of the bar indicates up- or downregulation of the underlying DEGs. The numbers of DEGs annotated with the enriched term are indicated at the end of each bar. Four key intersections are highlighted in dark grey (M *vs.* L only), amber (H *vs.* M only), red (H *vs.* L only) and purple (DEGs common between H *vs.* M and H *vs.* L contrasts).
